# Initial clinical experience of surface guided stereotactic radiation therapy with open-face mask immobilization for improving setup accuracy: a retrospective study

**DOI:** 10.1186/s13014-022-02077-4

**Published:** 2022-06-04

**Authors:** Shun Zhou, Junyu Li, Xianggao Zhu, Yi Du, Songmao Yu, Meijiao Wang, Kaining Yao, Hao Wu, Haizhen Yue

**Affiliations:** 1 Key Laboratory of Carcinogenesis and Translational Research (Ministry of Education/Beijing), Department of Radiation Oncology, Peking University Cancer Hospital and Institute, 52 Fucheng Road, Beijing, 100142 China; 2 Institute of Medical Technology, Peking University Health Science Center, 38 Huayuan Road, Beijing, 100191 China

**Keywords:** IGRT, SGRT, Stereotactic radiotherapy, Open face mask, Immobilization, Setup accuracy

## Abstract

**Purpose:**

To propose a specific surface guided stereotactic radiotherapy (SRT) treatment procedure with open-face mask immobilization and evaluate the initial clinical experience in improving setup accuracy.

**Methods and materials:**

The treatment records of 48 SRT patients with head lesions were retrospectively analyzed. For each patient, head immobilization was achieved with a double-shell open-face mask. The anterior shell was left open to expose the forehead, nose, eyes and cheekbones. The exposed facial area was used as region-of-interest for surface tracking by AlignRT (VisionRT Inc, UK). The posterior shell provided a sturdy and personalized headrest. Patient initial setup was guided by 6DoF real-time deltas (RTD) using the reference surface obtained from the skin contour delineated on the planning CT images. The endpoint of initial setup was 1 mm in translational RTD and 1 degree in rotational RTD. CBCT guidance was performed to derive the initial setup errors, and couch shifts for setup correction were applied prior to treatment delivery. CBCT couch shifts, AlignRT RTD values, repositioning rate and setup time were analyzed.

**Results:**

The absolute values of median (maximal) CBCT couch shifts were 0.4 (1.3) mm in VRT, 0.1 (2.5) mm in LNG, 0.2 (1.6) mm in LAT, 0.1(1.2) degree in YAW, 0.2 (1.4) degree in PITCH and 0.1(1.3) degree in ROLL. The couch shifts and AlignRT RTD values exhibited highly agreement except in VRT and PITCH (*p* value < 0.01), of which the differences were as small as negligible. We did not find any case of patient repositioning that was due to out-of-tolerance setup errors, i.e., 3 mm and 2 degree. The surface guided setup time ranged from 52 to 174 s, and the mean and median time was 97.72 s and 94 s respectively.

**Conclusions:**

The proposed surface guided SRT procedure with open-face mask immobilization is a step forward in improving patient comfort and positioning accuracy in the same process. Minimized initial setup errors and repositioning rate had been achieved with reasonably efficiency for routine clinical practice.

## Introduction

Stereotactic radiotherapy (SRT) is the delivery of three-dimensional dose distributions highly conformal to target volumes with sharp falloff to spare surrounding normal tissues. For brain tumors and head and neck (HN) tumors, SRT requires narrow margins to reduce toxicity to radiation-sensitive organs-at-risk (OARs) such as optic nerve, optic lens, parotid gland et al. [1, 2]. To guarantee the accuracy of dose delivery to tumor target, it is crucial to minimize patient positioning uncertainties, and issues in patient immobilization and setup workflow need to be carefully addressed to reduce initial setup errors and fraction-to-fraction variations [3, 4]. One of the commonly practiced positioning solutions applied for brain and HN patients is to use patient-specific thermoplastic masks for immobilization and on-board image guidance such as kV/MV portal imaging and cone beam computed tomography (CBCT) for tumor localization [5]. With the aid of online cross-modality registration, small setup errors can be easily identified by anatomy matching and then corrected by couch shift [6].

**Fig. 1:**
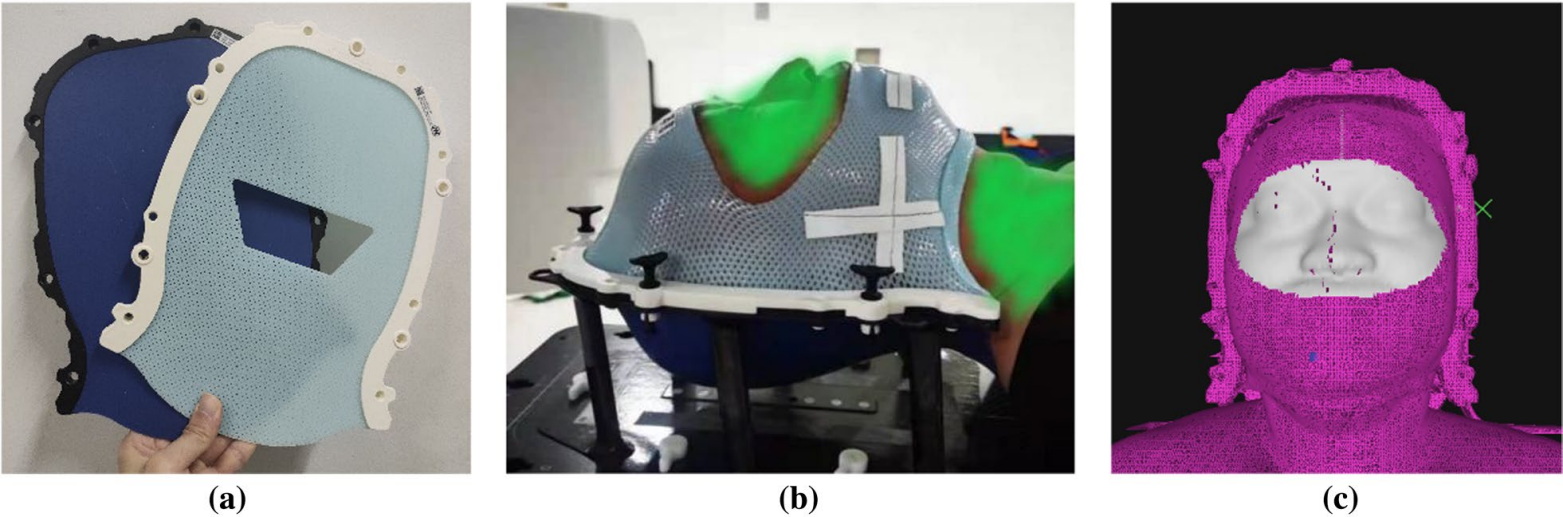
Open-face mask immobilization system used in this study: **a** the mask consisted of two shells, **b** illustration for typical patient immobilization, where the green shade is for anonymization, **c** typical reference surface in AlignRT, where the white area represents the ROI contour for surface tracking

However, the use of radiographic imaging has to be weighed against the health risk posed by additional imaging dose, which has prompted growing concerns [7–9]. Fortunately, optical surface imaging technology characterized by radiation-free has been developed, and the number of surface-guided radiation therapy (SGRT) systems commissioned into clinic has been rapidly increasing [10]. The typical surface guidance applications include patient positioning [11–13], motion monitoring [14–17] and breath tracking [18–20]. The SGRT system commissioned at our institution (Beijing Cancer Hospital, China) is the AlignRT (VisionRT Ltd, London, UK) as show in Fig. 1. A previous phantom-based study by Zhou et al. [21] evaluated the motion detection accuracy and isocenter congruence of the AlignRT system, finding that the system was able to identify sub-millimeter and sub-degree shifts and that the isocenter deviation with CBCT was negligible.

It is important to note that, in the pursuit of SGRT, the patient skin close to the tumor target has to be visible to AlignRT for track as region-of-interest (ROI), which poses a challenge to conventional closed masks. To circumvent this issue, open-face masks compatible with SGRT have been developed. Wiant et al. [14] and Mulla et al. [22] compared clinical performance between closed masks and open-face masks for HN radiotherapy. While the two studies were performed independently and the open-face masks involved were provided by two different vendors, identical conclusions were drawn: compared with closed masks, open-face masks provide comparable immobilization yet with reduced patient discomfort and anxiety. Nonetheless, when it comes to surface guided SRT for head lesion treatments, the studies related to open-face mask immobilization are quite limited. *Gregucci et al.* [23] *evaluated the clinical performance of the Solstice™ SRS immobilization system (CIVCO, FL, USA), which comprised of a head support, accuform cushion and open-face mask. At our institution, a different double-shell open-face immobilization system was adopted.* The purpose of this study is to evaluate the initial clinical experience of the surface guided SRT treatment procedure with open-face mask immobilization for improving setup accuracy.

## Materials and methods

As a retrospective study approved by the IRB, the clinical experience with 48 patients treated at our institution (Beijing Cancer Hospital, China) is presented. *Over the cohort, 30 patients were male and 18 were female. The patient ages ranged from 44 to 77 with the median as 63. The patients were with more than five brain metastases and received whole brain irradiation. CTV was the entire brain, and a 3-mm isotropic margin was added to define PTV. The prescription was 30 Gy /10 fractions.* All of the patients were immobilized with open-face masks and set up using the proposed surface guided setup workflow. The entire clinical chain from simulation to treatment as well as data analysis is detailed as below.

### Pretreatment

#### Patient immobilization

The open-face double shell positioning system (MacroMedics, Belgium) depicted in Fig. 1 was used at our institution for SRT patients. It consisted of two shells that were hosted on a dedicated support structure indexed onto the setup board. During mask molding, the anterior shell was left open to expose the face area, which included the nose, eyes and cheekbones. The exposed area can be used as ROI for AlignRT to track during optical surface imaging. The posterior shell, made of thick thermoplastic, was designed to offer a sturdy and personalized headrest that would improve patient comfort.

#### Patient simulation and planning

After the creation of open-face masks, patients were scanned on our CT-Sim (SOMATION Sensation Open, Siemens Healthineers, Germany). During simulation, four cross markers for localization were drawn on the tapes attached to the mask as shown in Fig. 1, three in the lower jawbones and one in the forehead. Once the simulation scan was finished, patient CT images were transferred to the Eclipse TPS (version 15.6, Varian Medical System Inc., USA) for structure contouring and treatment planning. Note that to provide the DICOM reference surface to AlignRT for surface tracking, the patient skinline named as BODY in TPS was first automatically extracted using an empirical predefined threshold of − 350 HU, and then carefully reviewed by physicist in case of any artifacts. Once approved by physicist and physician, treatment plans were transferred to linacs and AlignRT. At our institution, SRT treatments were all delivered on linacs using MLC-modulated RadipArc (Varian Medical System Inc., USA) with coplanar irradiation.

### Treatment

#### AlignRT surface guidance system

**Fig. 2:**
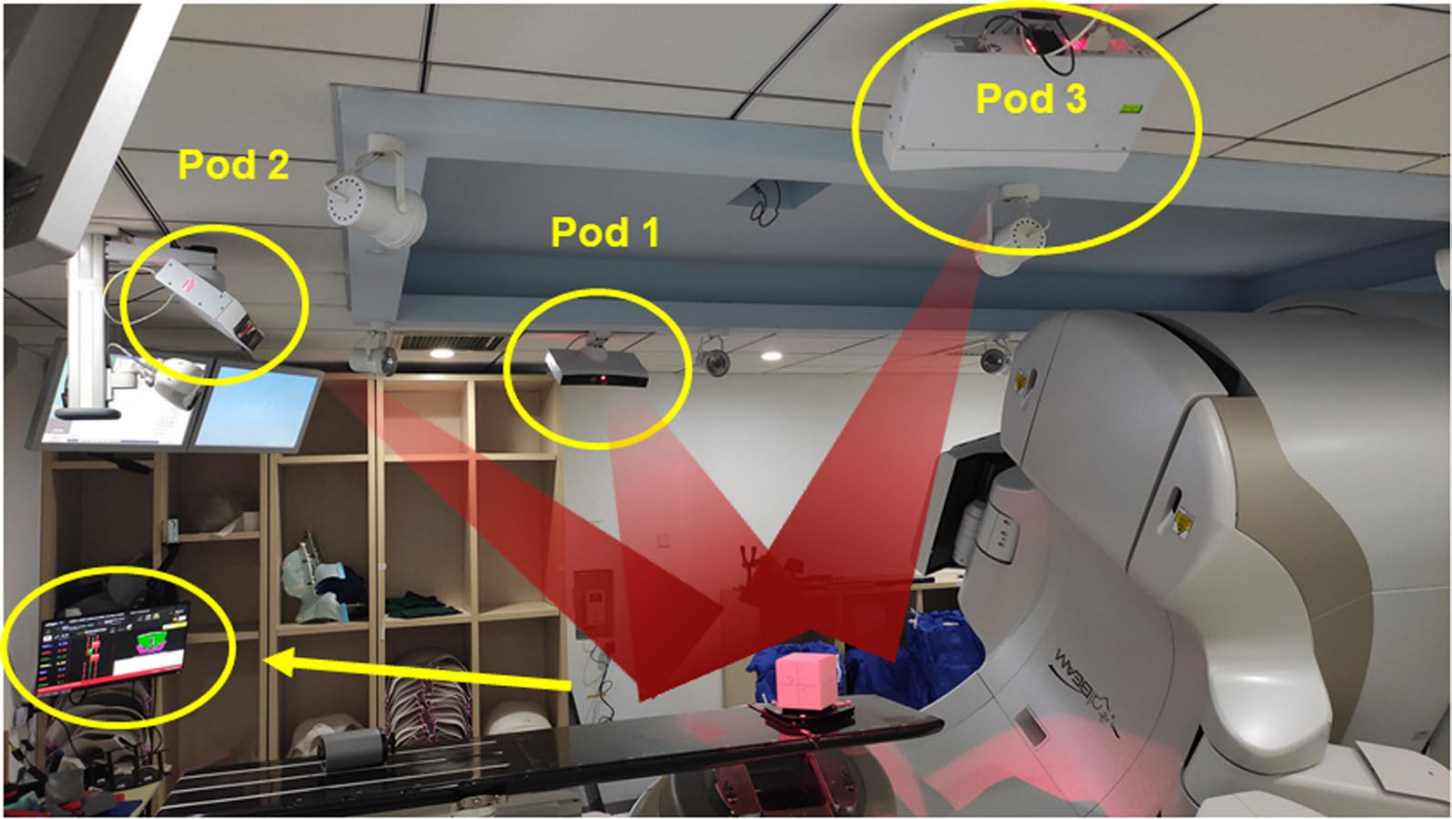
AlignRT surface imaging system integrated with a VitalBeam linac. The system consisted of two lateral pods and a rear pod. The surface displacement was represented in 6DoF real-time deltas (RTD)

The AlignRT surface guidance system at our institution was integrated with a VitalBeam (Varian Medical System Inc. USA) linac as shown in Fig. 2. The AlignRT system used three ceiling-mounted pods, each with a structured-light projector and two stereovision cameras. During surface imaging, projectors cast a speckle pattern onto the patient, and then the light pattern was captured by stereovision cameras to reconstruct the 3D real-time patient surface. The AlignRT software used a proprietary iterative closest points algorithm to register the real-time surface to the reference surface to derive relative displacement [10]. The derived surface displacement was displayed as six-degree-of-freedom (6DoF) real-time deltas (RTD) to indicate patient positioning errors.

Regular quality assurance is required for AlignRT performance maintenance. At our institution, daily QA, monthly QA, and isocenter calibration [24] as well as a self-developed weekly cube test [21] are periodically performed to ensure that the system works properly. The previous longitudinal study [21] shows that our AlignRT system is very stable and accurate to detect sub-millimeter and sub-degree shifts.

#### Patient setup and verification

**Fig. 3:**
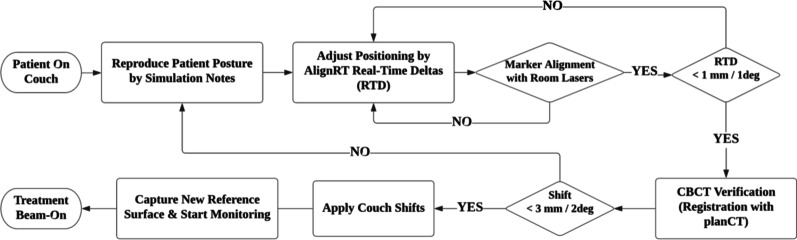
Institutional surface-guided patient setup workflow for SRT treatment

The standard operation procedure at our institution for surface guided SRT patient setup is in shown in Fig. 3. Patient-specific DICOM-RT files were first imported into AlignRT prior to treatment, and the patient record, plan tree, reference surface and ROI were created in Record mode. In this study, the BODY structure extracted from the planning CT was used as the reference surface for all fractions. The ROI for surface tracking was drawn to include the opening facial area (the nose, eyes, and cheekbones) and exclude any part of the thermoplastic mask.

On treatment days, the specific patient in AlignRT was retrieved and the *Treatment* mode was activated to guide patient setup. First, the mask support with the posterior shell was indexed onto the setup board, and the patient was asked to lie down and therapists adjusted his or her posture to fit the headrest. Second, the anterior shell was carefully placed and secured with screws to minimize unbalanced pressure. The alignment of localization marks to room lasers was confirmed in the same process. Third, therapists activated the real-time monitoring function in AlignRT and carefully tuned the patient position in reference to the displayed RTD till the RTD values were within the institutional tolerance, i.e., 1 mm in translation and 1 degree in rotation. Next, a CBCT scan and CBCT-to-planCT registration was performed to verify tumor localization and derive couch shifts for position correction. If the couch shift was within the institutional tolerance, i.e., 3 mm in any translation direction and 2 degree in any rotational direction, the shifts would be applied. Otherwise, patient repositioning was mandatory to perform. Once patient setup was confirmed, a new reference surface was captured and used for patient motion tracking in this fraction only. After all the above operations were finalized, treatment delivery was initiated, and real-time patient motion was monitored by AlignRT with beam-hold control.

### Recorded data and statistical analysis

Due to data missing in some cases, treatment records of 48 patients for 193 fractions in total were collected for this study, including: (a) RTD values in AlignRT at the end of patient initial setup; (b) setup corrections represented by CBCT couch shifts; (c) numbers of patient repositioning; (d) initial setup time, i.e., the time from patient lying down to therapists exiting the treatment room.

The AlignRT RTD values and CBCT setup corrections were first checked for normal distribution by Shapiro–Wilk test, and the results indicated normality was rejected (*p value* < 0.01) in several directions (CBCT-VRT, AlignRT-LNG, AlignRT-LAT, AlignRT-ROLL). Therefore, medians and percentiles of AlignRT RTD values and CBCT setup corrections were reported rather than means and standard deviations, and the difference in each direction was tested with the non-parametric Wilcoxon matched-pairs signed rank test. In this study, data analysis was performed in *OriginPro* (version 2021a, OriginLab, USA), and *p* value < 0.01 was considered statistically significant.

## Results

### Agreement of AlignRT RTD and CBCT couch shifts in translation

**Fig. 4:**
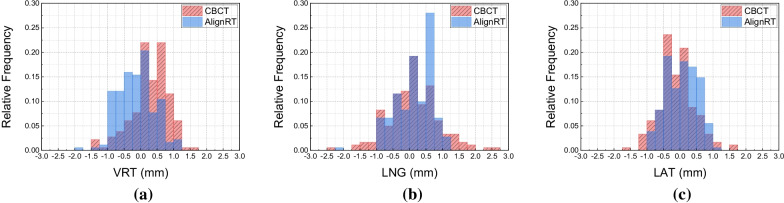
Histogram of AlignRT RTD and CBCT couch shifts distribution in **a** VRT, **b** LNG and **c** LAT

Table 1 lists the RTD values in AlignRT and couch shifts in CBCT in translational directions respectively. The median values of initial setups in RTD and couch shifts were quite close to zero, and the absolute translational setup errors of median 50% treatments (Q1 to Q3) were < 0.8 mm. Furthermore, the setup errors of all treatments fell within the action tolerance of ± 3 mm, and we did not any out-of-tolerance case that required repositioning. Figure 4 displays the distribution of translational RTD and couch shifts. As the p-values in Table 1 indicate, the level of overlapping between RTD and couch shifts in LNG (*p value* = 0.609) and LAT (*p value* = 0.015) were relatively larger than in VRT (*p value* < 0.01). We can also see that the cases requiring > 1.5 mm couch shifts were few, with only 9 in total.

**Table 1:** Translational displacements of AlignRT RTD and CBCT couch shifts

	VRT (mm)		LNG (mm)		LAT (mm)	
	*AlignRT*	*CBCT*	*AlignRT*	*CBCT*	*AlignRT*	*CBCT*
Median	− 0.1	0.4	0.2	0.1	0	− 0.2
Q1/Q3	− 0.6/0.2	0.1/0.7	− 0.3/0.5	− 0.5/0.7	− 0.4/0.4	− 0.4/0.1
Min/max	− 1.1/1	− 1.3/1.3	− 2.2/1.1	− 2.3/2.5	− 1/0.9	− 1.6/1.6
*p* value	< 0.01*		0.609		0.015	

*Level of statistical significance in this study: *p value* < 0.01

### Agreement of AlignRT RTD and CBCT couch shifts in rotation

**Fig. 5:**
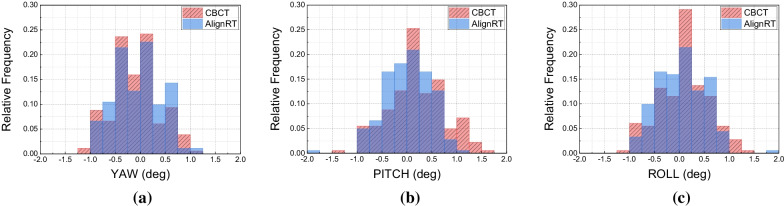
Histogram of AlignRT RTD and CBCT couch shifts distribution in **a** YAW, **b** PITCH and **c** ROLL

Table 2 shows the RTD values in AlignRT and couch shifts in CBCT in rotational directions respectively. The median values of initial setups in RTD and couch shifts were also quite close to zero, and the setup errors of median 50% treatments (Q1 to Q3) were < 0.6 degree. Moreover, the setup errors of all cases fell within the action tolerance of ± 2 degree, and we did not any out-of-tolerance case that required repositioning. The distribution of rotational RTD and couch shifts is shown in Fig. 5. As the *p* value in Table 2 indicates, the level of overlapping between RTD and couch shifts in YAW (*p value* = 0.628) and ROLL (*p value* = 0. 199) were relatively larger than in PITCH (*p value* < 0.01). In addition, we can see that the proportion of treatments that required > 1-degree couch shifts was minor, only 10.4% (20/193) in total.

**Table 2:** Rotational displacements of AlignRT RTD and CBCT couch shifts

	YAW (degree)		PITCH (degree)		ROLL (degree)	
	*AlignRT*	*CBCT*	*AlignRT*	*CBCT*	*AlignRT*	*CBCT*
Median	− 0.1	− 0.1	0	0.2	− 0.1	0.1
Q1/Q3	− 0.5/0.2	− 0.5/0.1	− 0.4/0.3	− 0.1/0.6	− 0.4/0.4	− 0.3/0.3
Min/Max	− 1/1	− 1.1/1.2	− 1.8/1.1	− 1.3/1.4	− 0.9/1.9	− 1/1.3
*p* value	0.628		< 0.01		0.199	

*Level of statistical significance in this study: *p* value < 0.01

### Repositioning rate and setup time

As shown in Tables 1 and 2, the maximal absolute couch shifts are 2.5 mm in translation and 1.9 degrees in rotation, which are within our institutional action tolerance for repositioning, i.e., 3 mm in any translational shift and 2 degrees in any rotational shift. As a result, none of the treatments require repositioning of the patient in terms of out of tolerance. Nevertheless, according to the treatment records, repositioning was eventually performed in seven treatments at the request of the therapist/physicist/physician somehow.

Since this work was performed retrospectively, only the last 39 treatments were retrievable for CCTV records. In these cases, the initial setup time ranged from 52 to 174 s, with a mean and median of 97.72 s and 94 s respectively.

## Discussion

The advantages of the optical surface imaging over X-ray imaging are radiation-free and real-time response, and the clinical performance of SGRT systems has been investigated in many tumor sites [12]. However, relevant work for head lesion treatment was few, and a standardized clinical operation procedure has not been established yet, partially due to the usage of closed masks. In this study, we share our initial clinical experience using highly customized open-face mask immobilization in conjunction with surface guidance for stereotactic treatment of 48 head tumor patients. The step-by-step workflow from simulation to treatment was detailed, and the initial setup errors derived from CBCT guidance were statistically analyzed. Compared with previous studies [14, 16, 22, 25], the proposed surface guided setup procedure achieved the highest setup accuracy of < 3 mm in all translation directions and < 2-degree in rotational directions.

The improved setup accuracy can be attributed to three aspects. First, the sturdy and comfortable double-shell open-face masks exhibited excellent immobilization performance in reproducing patient positions. Second, the proposed workflow maximized the utility of surface guidance in real-time imaging. The surface displacement represented in 6DoF RTD effectively facilitated therapists in fine-tuning patient postures. Third, the endpoint requirement of < 1 mm and < 1 degree in RTD for surface guided setup was essential in minimizing couch shifts in CBCT guidance. This can further explain that we did not identify any case that required mandatory patient repositioning.

Meanwhile, it is worth noting that, despite the endpoint requirement of < 1 mm and < 1 degree in AlignRT RTD, there were still a few outlier cases. These cases indicate that when the patients were initially set up, they moved voluntarily and subtly. This patient motion issue, especially why patients move, will be investigated in further work.

As for clinical efficiency, all of the setups in this study were finished within 3 min, and we did not identify any case that were out of action-level tolerance (3 mm /2 degree) that required repositioning. Since less time was required in patient setup, we believe that the overall treatment throughout was eventually increased. This treatment efficiency is very significant in terms of optimizing clinical resource utilization, and future work will follow to address this issue.

As for limitations of this study, first, stereotactic radiosurgery (SRS) patients were not enrolled in the cohort, owing to our institutional practice that all SRS treatments were assigned to an Edge Radiosurgery System (Varian Medical System Inc., USA) rather than the VitalBeam linac used for this study. Despite this, we do believe that the proposed procedure is applicable to surfaced guided SRS treatments. Second, this study was based on our initial clinical experience with open-face mask immobilization in combination with surface guided patient setup. As our experience in AlignRT and open-face masks grows, the streamlined procedure may be further optimized. Third, the issue of intra-fraction patient motion was not addressed herein. While we received alerts of beam-holding during beam delivery, the case number was too small to make any reasonable conclusion. Further updated work of a larger cohort study with optimized workflow and intra-fraction patient motion analysis is expected in future. *In addition, it is important to note, while the use of SGRT is a topic of growing interest in modern radiotherapy, our experience indicates that at the moment SGRT is complimentary to CBCT rather than an alternative to replace it.*

## Conclusions

In summary, we have established a surface guided SRT procedure with double-shell open-face mask immobilization, which is a step forward in enhancing patient comfort and positioning accuracy in the same process. The clinical experience was retrospectively reviewed, and procedure has proven to be reasonably efficient with minimized initial setup errors and repositioning rate for routine clinical application. Further updated work in a larger cohort will follow to evaluate the clinical performance in intra-fraction patient motion.

## Data Availability

The data that support this study are not openly available due to ethical and privacy concerns and are available from the corresponding author upon reasonable request.
